# Supplementary material for: Quantification of sporozoite expelling by Anopheles mosquitoes infected with laboratory and naturally circulating P. falciparum gametocytes
Source: eLife. 2024 Mar 22;12:RP90989. doi: 10.7554/eLife.90989 (PMC10959522; doi:10.7554/eLife.90989)
Supplement: Figure 3—source data 1. [file elife-90989-fig3-data1.docx]

| Figures | Only including observations with: | Spearman’s correlation |
| --- | --- | --- |
| Fig. 3B | <5 oocysts sheets | 0.50 (95% CI: 0.32, 0.64, p<0.0001) |
| Fig. 3B | <10 oocysts sheets | 0.64 (95% CI: 0.52, 0.73, p<0.0001) |
| Fig. 3B | <20 oocysts sheets | 0.71 (95% CI: 0.61, 0.78, p<0.0001) |
| Fig. 3C | SPZ<10,000 | 0.29 (95% CI: -0.07, 0.58, p=0.1094) |
| Fig. 3C | SPZ<50,000 | 0.29 (95% CI: 0.08, 0.47, p=0.0086) |
| Fig. 3C | SPZ<100,000 | 0.33 (95% CI: 0.14, 0.5, p=0.0011) |
| Fig 3. Fig. suppl. 1 | SPZ<10,000 | 0.24 (95% CI: -0.04, 0.48, p=0.0891) |
| Fig 3. Fig. suppl. 1 | SPZ<50,000 | 0.19 (95% CI: 0.02, 0.36, p=0.0280) |
| Fig 3. Fig. suppl. 1 | SPZ<100,000 | 0.13 (95% CI: -0.03, 0.29, p=0.1000) |

**Figure 3 - Source data 1. Re-analysis of major correlations using ranges of observed oocyst sheets and salivary gland sporozoite loads.** Analyses were repeated to examine whether correlations lost statistical significance when a narrower range of oocyst or sporozoite densities was included. The table shows the cut-offs for maximum oocyst numbers (e.g. <5, <10, <20) and sporozoite numbers (e.g. <10,000; <50,000; <100,000) to determine how correlations hold across the entire range of observed oocyst sheets and salivary gland sporozoite load. Correlation values and confidence intervals are rounded to two decimal places.
