# Supplementary material for: Quantification of sporozoite expelling by Anopheles mosquitoes infected with laboratory and naturally circulating P. falciparum gametocytes
Source: eLife. 2024 Mar 22;12:RP90989. doi: 10.7554/eLife.90989 (PMC10959522; doi:10.7554/eLife.90989)
Supplement: Figure 4—source data 1. [file elife-90989-fig4-data1.docx]

| Figures | Only including observations with: | Spearman’s correlation |
| --- | --- | --- |
| Fig. 4B | <5 oocysts sheets | 0.83 (95% CI: 0.54, 0.95, p=0.0002) |
| Fig. 4B | <10 oocysts sheets | 0.72 (95% CI: 0.40, 0.89, p=0.0005) |
| Fig. 4B | <20 oocysts sheets | 0.80 (95% CI: 0.57, 0.91, p<0.0001) |
| Fig. 4C | SPZ<10,000 | 0.74 (95% CI: 0.42, 0.89, p=0.0003) |
| Fig. 4C | SPZ<50,000 | 0.62 (95% CI: 0.34, 0.80, p=0.0002) |
| Fig. 4C | & SPZ<100,000 | 0.63 (95% CI: 0.38, 0.79, p<0.0001) |

**Figure 4 – Source data 1. Re-analysis of major correlations using ranges of observed oocyst sheets and salivary gland sporozoite loads.** Analyses were repeated to examine whether correlations lost statistical significance when a narrower range of oocyst or sporozoite densities was included. The table shows the cut-offs for maximum oocyst numbers (e.g. <5, <10, <20) and sporozoite numbers (e.g. <10,000; <50,000; <100,000) to determine how correlations hold across the entire range of observed oocyst sheets and salivary gland sporozoite load. Correlation values and confidence intervals are rounded to two decimal places.
